# Unraveling Ferroptosis: A New Frontier in Combating Renal Fibrosis and CKD Progression

**DOI:** 10.3390/biology14010012

**Published:** 2024-12-27

**Authors:** Rui Jin, Yue Dai, Zheng Wang, Qinyang Hu, Cuntai Zhang, Hongyu Gao, Qi Yan

**Affiliations:** 1 Department of Geriatrics, Tongji Hospital, Tongji Medical College, Huazhong University of Science and Technology, Wuhan 430030, China; jr23hust@hust.edu.cn (R.J.); dy4987325@163.com (Y.D.); wangzheng19960330@163.com (Z.W.); qy6049dwyy@163.com (Q.H.); ctzhang0425@163.com (C.Z.); 2 Key Laboratory of Vascular Aging, Ministry of Education, Tongji Hospital, Tongji Medical College, Huazhong University of Science and Technology, Wuhan 430030, China; 3 Hubei Provincial Clinical Medical Research Center for Nephropathy, Enshi 445000, China

**Keywords:** ferroptosis, cell death, molecular mechanism, chronic kidney disease (CKD), renal fibrosis

## Abstract

Chronic kidney disease (CKD) is a global health concern caused by conditions such as hypertension, diabetes, hyperlipidemia, and chronic nephritis, leading to structural and functional kidney injury. Kidney fibrosis is a common outcome of CKD progression, with abnormal fatty acid oxidation (FAO) disrupting renal energy homeostasis and leading to functional impairments. This results in maladaptive repair mechanisms and the secretion of profibrotic factors, and exacerbates renal fibrosis. Understanding the molecular mechanisms of renal fibrosis is crucial for delaying CKD progression. Ferroptosis is a type of discovered an iron-dependent lipid peroxidation-regulated cell death. Notably, Ferroptosis contributes to tissue and organ fibrosis, which is correlated with the degree of renal fibrosis. This study aims to clarify the complex mechanisms of ferroptosis in renal parenchymal cells and explore how ferroptosis intervention may help alleviate renal fibrosis, particularly by addressing the gap in CKD mechanisms related to abnormal lipid metabolism under the ferroptosis context. The goal is to provide a new theoretical basis for clinically delaying CKD progression.

## Simple Summary

CKD is a common outcome of the progressive deterioration caused by various primary and secondary kidney diseases. It is characterized by renal interstitial fibrosis and a progressive decline in renal function. As CKD advances, patients often experience disturbances in iron and lipid metabolism, along with inflammation and oxidative stress, leading to tubular epithelial cell death. Ferroptosis, an iron-dependent form of cell death driven by lipid peroxidation, plays a crucial role in tissue and organ fibrosis. This review highlights the potential mechanisms by which ferroptosis promotes renal fibrosis and explores the therapeutic potential of targeting ferroptosis to treat kidney fibrosis.

## 1. Introduction

**Figure 1:**
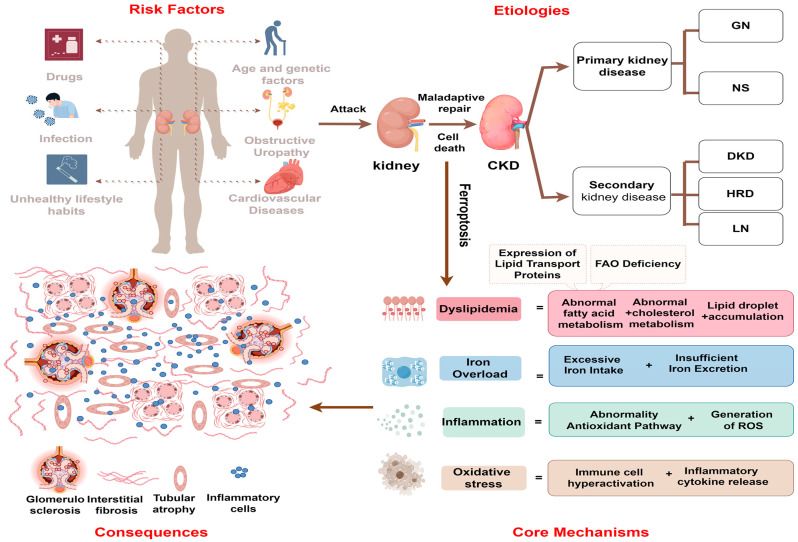
The causes and consequences of ferroptosis in cells during CKD. Various risk factors such as aging, infection, and the prolonged toxic effects of nephrotoxic drugs disrupt renal energy metabolism, damage the immune barrier, and lead to metabolic disorders in renal parenchymal cells. This results in iron overload, lipid accumulation, inflammation, and oxidative stress, collectively activating ferroptosis, which spreads among renal parenchymal cells, damaging tubular structure and function. The maladaptive repair process leads to glomerulosclerosis, interstitial fibrosis, and increased hyperfiltration pressure. GN, glomerular nephritis; NS, nephrosis syndrome; DN, diabetic nephropathy; HRD, hypertensive renal disease; LN, lupus nephritis.

CKD is a leading cause of mortality worldwide, affecting 15–20% of adults worldwide [1]. Primary and secondary nephropathies are the major contributors of chronic kidney injury, with the persistent synthesis and accumulation of fibrous matrix in the interstitial space leading to extensive scarring of the kidneys. This results in tubular interstitial fibrosis and glomerulosclerosis, accelerating the progression to end-stage renal disease (ESRD) [2]. CKD also causes a range of complications, including cardiovascular diseases, anemia, metabolic acidosis, and disruptions in bone mineral metabolism. Under chronic stimulation, several types of programmed cell death are triggered, including ferroptosis. The cellular release of damage-associated molecular patterns (DAMPs) is promoted by ferroptosis, which contributes to the progression of CKD (Figure 1).

Ferroptosis is an iron-dependent, nonapoptotic form of regulated cell death (RCD) [3]. It is characterized by decreased activity of glutathione peroxidase 4 (GPX4), reduced antioxidant capacity, the accumulation of lipid reactive oxygen species (ROS), and the consumption of polyunsaturated fatty acids (PUFAs) in the plasma membrane. Morphologically, ferroptotic cells exhibit smaller mitochondria, an increased membrane density, and reduced cristae morphologically [3]. Ferroptosis is involved in the pathogenesis of fibrotic diseases. The imbalance of intracellular iron and redox homeostasis during ferroptosis disrupts the secretion of transforming growth factor-beta (TGF-β) in the fibrotic process. Simultaneously, ferroptosis increases intracellular free iron, which generates more ROS and exacerbates fibrosis [4]. Iron levels are correlated with the extent of fibrosis and kidney atrophy, and early noninvasive methods, such as magnetic resonance imaging (MRI), can be used to assess iron content in fibrotic kidneys, enhancing CKD diagnosis [5]. In fibrotic kidney injury, proximal tubule cells express proinflammatory and profibrotic factors, with ferroptosis acting as a critical susceptibility pathway [6]. Furthermore, pharmacologically inducing ferroptosis can hinder the progression of renal fibrosis and promote adaptive repair in damaged cells [7,8,9,10]. These findings highlight the pivotal role of ferroptosis in the progression in renal fibrosis. This review aims to elucidate the molecular mechanisms underlying ferroptosis, focusing on the links between ectopic lipid deposition, lipotoxicity, and ferroptosis in CKD, and provides new insights for future CKD treatment strategies.

## 2. General Mechanisms of Ferroptosis

**Figure 2:**
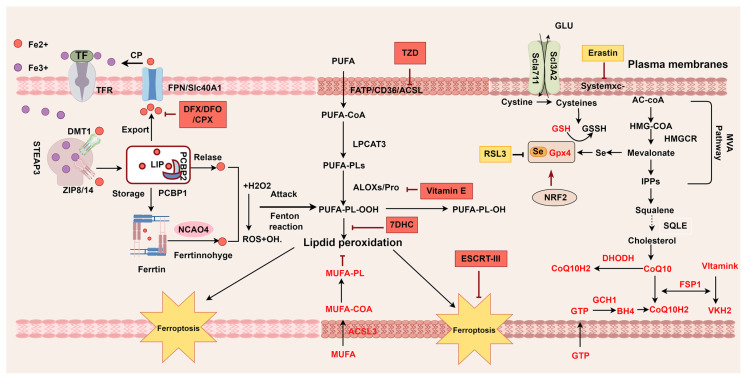
Mechanisms of ferroptosis. Ferroptosis is characterized by imbalances between oxidative and antioxidant pathways. Iron overload and PUFAs drive the production of oxidized lipids, which are key contributors to ferroptotic cell death. Several regulatory factors and monounsaturated fatty acids (MUFAs) play crucial roles in counteracting oxidative stress and providing antioxidant protection. TF transferrin; TBI transferrin-bound iron; TfR transferrin receptor; ZIP Zrt- and Irt-like family proteins; DMT1 divalent metal transporter 1; FTH ferritin heavy; FTL ferritin light; STEAP3 endosomal ferrireductase 3; PCBP iron chaperones of the poly-rC-binding protein family; FPN ferroportin; NCOA4 nuclear coactivator 4; NRF2 nuclear factor erythroid2-related factor 2; LIP labile iron pool; MUFA monounsaturated fatty acid; PUFA polyunsaturated fatty acid; FATP fatty acid transport protein; CD36 CD36 molecule; ACSL acyl-CoA synthetase long-chain family member; LPCAT3 Lysophosphatidylcholine Acyltransferase 3; NOXs NADPH oxidases; ALOX Lipoxygenase; SLC7A11 solute carrier family 7 member 11; SLC3A12 solute carrier family 3 member 2; Glu glutamic acid; GPX4 glutathione peroxide 4; GSH glutathione; GSSG glutathione disulfide; GTP guanosine triphosphate; DHODH dihydroorotate dehydrogenase; LOOH lipid hydroperoxides; LOH lipid alcohols; FSP1 ferroptosis suppressor protein 1; GTP Guanosine triphosphate;BH4 tetrahydrobioptrin; GCH1 GTP cyclohydroxylase1; IPP isopentenyl-pyrophosphate; MVA; mevalonatepath; HMG-CoA 3-hydroxy-3-methylglutaryl CoA; DFX Deferasirox; DFO Deferoxamine; CPX ciclopirox; TZD Thiazolidinediones; 7-DHC 7-dehydrocholesterol. This picture was drawn by Figdraw 2.0.

This section provides an overview of the oxidative and antioxidant pathways involved in the mechanisms of ferroptosis, highlighting the key regulatory molecules involved (Figure 2). Research into the mechanisms of ferroptosis has enhanced our understanding of this process.

### 2.1. Driving Ferroptosis: Iron Overload

Iron overload initiates ferroptosis by driving redox reactions under pathological conditions. Even subtle changes in intracellular iron levels can affect iron homeostasis. Transferrin (TF) captures extracellular iron and binds to transferrin receptors (TFRs) to deliver transferrin-bound iron (TBI) into cells [11,12]. TFR1 serves as a biomarker that distinguishes ferroptosis from other forms of cell death [13]. When the endosomal pH decreases to 5.5–6.5, Fe^3+^-TF is converted to Fe^2+^ by six-transmembrane epithelial antigen of prostate 3 (STEAP3) and transported to the cytoplasm via metal-ion transporter-1 (DMT1/SLC11A2) or ZRT/IRT-like protein (ZIP) 8 or 14, forming a labile iron pool (LIP). Chaperones like Poly (rC)-binding protein (PCBP) regulate Fe^2+^ in the LIP and bind glutathione with low affinity to maintain iron homeostasis [14,15,16]. Excess iron is stored in ferritin, which consists of two subunits, FTH (which oxidizes Fe^2+^ to Fe^3+^) and FTL (which aids in iron mineralization and core formation) [3]. Nuclear coactivator 4 (NCOA4) selectively mediates ferritinophagy in the lysosome, releasing Fe^2+^ and triggering ferroptosis [17,18,19,20]. The only cellular iron export pathway is regulated by hepcidin through Ferroportin (FPN/SLC40A1) [21,22]. Hepcidin, a hormone synthesized by the liver, binds to and degrades Ferroportin (FPN), reducing intracellular iron efflux [23,24]. The exported Fe^2+^ is then oxidized to Fe^3+^ by ceruloplasmin (CP), allowing it to re-enter the intracellular iron cycle.

### 2.2. Ferroptosis Execution: Formation of Lipid Peroxidation Substrates

PUFAs are the primary targets of lipid peroxidation, and their accumulation of lipid peroxides ultimately triggers ferroptosis [25]. The intake, metabolism, and synthesis of fatty acids, phospholipids, and cholesterol are critical processes, with arachidonic acid (AA) and adrenic acid (AdA) being key contributors to ferroptosis [26]. AA and AdA are acylated by acyl-CoA synthetase long-chain family member 4 (ACSL4) and esterified by lysophosphatidylcholine acyltransferase 3 (LPCAT3) to form AA/AdA-PE [27]. These molecules are then oxidized by lipoxygenase (LOX) or cytochrome P450 reductase (POR) to generate AA/AdA-PE-OOH [28,29]. In contrast, ACSL3 acylates monounsaturated fatty acids (MUFAs), displacing PUFAs in cell membranes and reducing lipid peroxidation [30]. The balance between ACSL4-PUFAs and ACSL3-MUFAs plays a crucial role in determining membrane stability [31,32,33].

### 2.3. Accelerating Ferroptosis: Imbalance in Antioxidant Pathways

#### 2.3.1. Classical Antioxidant Pathway: System Xc-GSH—GPX4

The antioxidant system is crucial for cellular redox reaction balance, with the system Xc-GSH-GPX4 axis serving as the earliest and key regulator in halting ferroptosis. This system is fundamental to antioxidant defense. System Xc- consists of two subunits: SLC7A11 (also known as xCT) and SLC3A2. Its activity and expression are influenced by external factors. For instance, the activation of the TP53 gene downregulates SLC7A11 expression, while elevated extracellular glutamate levels inhibit cysteine uptake via System Xc [34,35,36,37]. L-cysteine, a key amino acid, limits the synthesis of GSH and GPX4 [3], with selenocysteine being an essential amino acid for the active site of GPX4. The inhibition of the mevalonate pathway impairs selenocysteine tRNA maturation, disrupting GPX4 synthesis [38]. GSH acts as a reducing substrate for GPX4 and a free radical scavenger. GPX4 is critical for protecting cells from ferroptosis. NRF2 regulates antioxidant gene expression to maintain redox balance. It can upregulate GPX4 expression, directly or indirectly, protecting cells from ferroptosis [39,40,41,42]. NRF2 also activates System Xc- to increase intracellular cysteine levels and modulates the mTORC1/4EBP1/GPX4 pathway to enhance GPX4 synthesis and reduce its degradation [43]. Focusing on NRF2 agonists, which activate the Nrf2/GPX4 signaling pathway to inhibit ferroptosis, could be a promising future therapeutic target in CKD. The subcellular localization of different GPX4 isoforms (cytoplasm, nucleus, and mitochondria) determines their specific functions. In conclusion, the system Xc-GSH-GPX4 pathway is intricately involved in the sophisticated antioxidant defense system that regulates ferroptosis.

#### 2.3.2. Parallel Antioxidant Pathway: FSP1-CoQ10-NAD(P)H

Metabolic pathways parallel to GPX4 also contribute to the mitigation of ferroptosis. Ferroptosis suppressor protein 1 (FSP1) plays a crucial role in preventing ferroptosis independently of GPX4 via the FSP1-CoQ10-NAD(P)H pathway [44,45]. FSP1, located in lipid droplets and the plasma membrane, utilizes NADPH to convert coenzyme Q10 (CoQ10) into ubiquinol, which neutralizes oxygen free radicals and reduces ferroptosis [46]. CoQ10, essential for mitochondrial electron transfer, regenerates α-tocopherol, which scavenges free radicals and prevents ferroptosis.

Research also shows that FSP1 protects against ferroptosis by enhancing cell membrane repair through an ESCRT-III-FSP1 mechanism, independent of CoQ10 [47,48,49]. Additionally, various forms of vitamin K, including phylloquinone, MK4, and menadione, inhibit ferroptosis in mouse embryonic fibroblasts and protect against ischemia–reperfusion injury in liver and kidney models [50,51]. 

GTP cyclohydrolase I (GTPCH-I), the rate-limiting enzyme in the synthesis of BH4, plays a protective role by scavenging oxidants [52,53]. BH4 strongly counteracts ferroptosis induced by inducers and GPX4 knockout [27], making the GCH1-BH4 pathway a key anti-ferroptosis by selectively preventing phospholipid depletion. The pathways described above are involved in ferroptosis, and a deeper investigation into the intricate molecular mechanisms underlying these processes is warranted.

#### 2.3.3. Mitochondrial Antioxidant Pathway

Mitochondrial dysfunction, due to impaired antioxidant pathways, is one of the earliest observed organelle changes observed during ferroptosis, underscoring its critical role in this process. DHODH catalyzes the reduction in ubiquinone (CoQ) to dihydroubiquinone (CoQH2), which acts as an antioxidant by preventing the formation of lipid peroxides in the cell membrane, thus inhibiting ferroptosis. Recent research indicated that mitochondrial DHODH and GPX4 collaborate to suppress ferroptosis. Inhibiting DHODH with Brequinar (BQR) induces ferroptosis in cells with low GPX4 expression, increasing their susceptibility to ferroptosis [54,55]. Moreover, combining BQR with the system Xc- inhibitor sulfasalazine effectively halts cancer cell proliferation, irrespective of their GPX4 levels [56]. The DHODH-CoQH2 pathway operates independently of cytoplasmic GPX4 or FSP1, suggesting that further investigation into other subcellular compartments may reveal additional mechanisms for ferroptosis defense.

## 3. The Foundation of Ferroptosis in CKD

The activation of ferroptosis is closely associated with key pathophysiological processes in CKD, including inflammation, immune cell accumulation, and lipid metabolism disorders. These contribute to sustained renal damage and dysfunction, facilitating the transition from AKI to CKD and ultimately leading to irreversible fibrosis [57]. Preventing fibrosis is crucial for slowing CKD progression. The pathogenic mechanisms underlying fibrosis in CKD are sophisticated, and emerging research suggests a strong link between ferroptosis and the development of renal fibrosis.

### 3.1. Inflammation Trigger for CKD

Inflammation is crucial in kidney repair, leading to tubular damage, fibrosis, and CKD progression [58,59]. Continuous damage to immune cells and renal parenchymal cells attracts inflammatory cells and drives the release of cytokines, chemokines, and adhesion molecules [60,61]. This section explores how immune and renal parenchymal cell interactions during inflammation trigger CKD mechanisms, highlighting the potential pathways of ferroptosis in CKD.

#### 3.1.1. Activate of Innate Immune Cells

Within the first 48 h, necrotic renal tubular epithelial cells (TECs) release DAMPs and ROS, which are recognized by macrophage receptors, leading to M1 polarization and the production of proinflammatory cytokines such as TNF-α, IL-1β, and IL-6. After 48 h, macrophages shift to the M2 phenotype, where they secrete repair-promoting cytokines (IL-10 and IL-1Rα) and profibrotic factors (TGF-β1, FGF-2, PDGF, and MMP-9) that activate fibroblasts during renal injury. This shift fosters myofibroblast proliferation in the renal interstitium, contributing to glomerular and tubular capillary damage [62,63]. Renal fibrosis is largely driven by imbalanced M1/M2 macrophage polarization and persistent inflammation.

Dendritic cells (DCs) play a dual role in renal inflammation. On one hand, they recruit T cells and mediate the release of toxic mediators, leading to acute tubular interstitial damage and chronic glomerulonephritis. On the other hand, they promote the activation of regulatory T cells, resolving the immune response and facilitating renal repair [64].

Neutrophils respond swiftly to renal injury by interacting with endothelial chemokines and adhesion molecules during the initial injury stages. In a unilateral ureteral obstruction (UUO) mouse model, neutrophil depletion reduced inflammatory factor expression and inhibited kidney fibrosis [65].

T cells are central to inflammation, and the pathogenic role of CD4^+^ T cells has been identified in the UUO model [66]. In mouse models of ischemia and nephrotoxicity, CD8^+^ T cell deficiency provided less protection compared to CD4^+^ T cell deficiency [67,68].Activated CD4^+^ T cells differentiate into various subsets, each with distinct effects [69]. Treg cells stimulated by IL-2 and IL-33 help mitigate persistent renal injury [70]. Furthermore, immunosuppressants such as sirolimus and mycophenolate mofetil have been demonstrated to reduce renal fibrosis in animal models of obstructive nephropathy [71,72,73,74].

B cells are involved in various immune processes, and research on B cells in renal fibrosis remains limited. The initial studies highlighted B cell interactions with other immune cells during renal injury, potentially driving the progression from AKI to CKD. Early B cell infiltration is linked to macrophage accumulation and acute inflammation in UUO. Depleting B cells reduces macrophage infiltration and renal fibrosis, highlighting the significant role of B cells in renal fibrosis development [75]. Prolonged B cell targeting could improve renal function and impede the progression of CKD [76].

#### 3.1.2. Maladaptive Renal Repair

TECs are particularly vulnerable to external injury. While mild damage is often repairable, severe or persistent injury activates pattern recognition receptors in TECs. This triggers the release of high mobility group box protein 1 (HMGB1), which stimulates Toll-like receptor 4 (TLR4) in TECs, leading to NF-κB pathway activation and the production of inflammatory factors such as IL-6, TNF-α, and MCP-1 [77]. TECs also secrete chemokines like MCP-1, recruiting macrophages and mesangial cells to produce TGF-β1 in renal failure [78]. If TECs lose their regenerative, irreversible basement membrane, thickening and interstitial fibrosis occur.

During injury, endothelial cells recruit leukocytes, promoting immune cell adhesion, which exacerbates ischemia and tubular injury [79,80,81]. Acute inflammation can transition to chronic inflammation [82], prompting endothelial cells to express chemokines and adhesion molecules in response to the local inflammatory environment [82,83]. Persistent inflammation causes local endothelial cell proliferation, and some undergo endothelial-to-mesenchymal transition, driving renal fibrosis progression [80].

Mesangial cells release proinflammatory factors, including TNF-α and IL-6, along with oxidants and growth factors in kidney injury [84]. This promotes mesangial cell proliferation, alters endothelial permeability, and increases extracellular matrix (ECM) production, ultimately leading to glomerulosclerosis [85,86]. Damage to podocytes not only triggers inflammatory responses but also disrupts the filtration barrier, leading to proteinuria and tubular degeneration, which further worsen kidney dysfunction [87].

### 3.2. Ectopic Lipid Deposition

**Figure 3:**
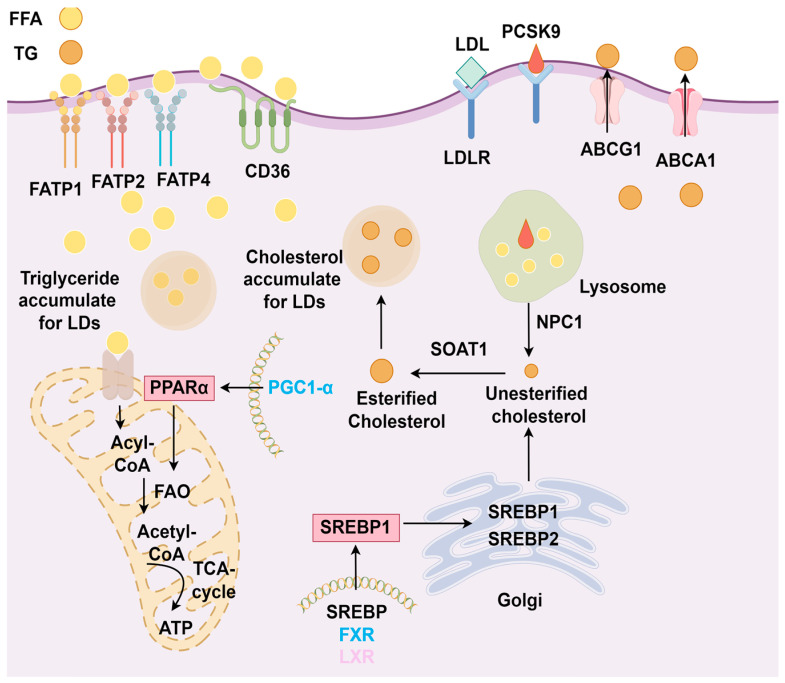
Physiological mechanisms of lipid metabolism in the kidney. In proximal tubular cells, FATP and CD36 on podocytes take up free fatty acids (FFAs) from the bloodstream for fatty acid oxidation (FAO) to produce ATP. Peroxisome proliferator-activated receptor α (PPARα), PPARγ coactivator 1α (PGC-1α), and AMP-activated protein kinase (AMPK) regulate FAO. Excess FAs accumulate and are stored as lipid droplets. Liver X receptor (LXR) and farnesoid X receptor (FXR) regulate the expression of sterol regulatory element-binding protein 1 (SREBP1). SREBP1 and SREBP2 are transported from the endoplasmic reticulum to the Golgi apparatus, where they are cleaved and then transported to the nucleus to synthesize unesterified cholesterol. This cholesterol is subsequently converted into esterified cholesterol (CE) by sterol O-acyltransferase 1 (SOAT1) or transported to the plasma membrane by ATP-binding cassette transporters A1 (ABCA1) and G1 (ABCG1) to form low-density lipoprotein (LDL) and high-density lipoprotein (HDL). Proprotein convertase subtilisin/kexin type 9 (PCSK9) binds and degrades LDL to maintain cholesterol metabolism balance. The accumulation of free cholesterol (FC) results in the formation of lipid droplets, maintaining normal levels of fatty acids and cholesterol in the kidney.

Understanding the process of lipid metabolism in a healthy kidney is crucial for exploring lipid-related kidney damage (Figure 3). Excessive and unprocessed lipids accumulation, characterized by elevated triglycerides (TGs) and decreased FAO, leads to organelle dysfunction, cell injury, oxidative stress, autophagy dysregulation, and inflammation—a condition known as lipid nephrotoxicity [88]. This condition disrupts energy metabolism and is a key driver of renal fibrosis. The following sections examine the impact of fatty acid and cholesterol toxicity on CKD.

#### 3.2.1. Fatty Acid Toxicity

Fatty acid homeostasis is an important component in kidney lipid metabolism. Renal tubules, rich in mitochondria, rely on FAs for energy under physiological conditions. Under low-glucose conditions, endothelial and mesangial cells demand FAO for energy.

Defective FAO contributes to the progression of renal fibrosis. The overexpression of the key upstream FAO transcription factor PGC-1α in TECs restores the FAO rate-limiting enzyme CPT1, reducing fibrosis induced by folic acid [89].The PPARα agonist fenofibrate protects against interstitial fibrosis in a mouse model of ischemia–reperfusion injury [90]. AMP-activated protein kinase (AMPK), a key upstream signaling molecule, also regulates the expression of CPT-1α. Studies have shown that AMPK activity is associated with kidney lipid accumulation and lipotoxicity. In a rat model of non-diabetic renal interstitial fibrosis, AMPK activation corrects FAO dysfunction in TECs and improves kidney fibrosis [91].

FAs are transported into cells mainly through fatty acid transport proteins (FATP1, FATP2, and FATP4), while epithelial and mesangial cells utilize CD36 for FA uptake. Podocytes primarily take up FA through CD36. Palmitic acid treatment increases CD36 levels in podocytes, causing intracellular lipid accumulation and ROS production, mitochondrial dysfunction, and elevated profibrotic factors, ultimately contributing to glomerular injury [92]. Proximal tubules depend on FAO for energy, while CD36 and FATP2 facilitate FA uptake and transport. In a UUO mouse model, the inhibition or knockout of FATP2 in proximal TECs (PTECs) prevents lipotoxicity [93]. In 8-week-old mice, FA accumulation is observed, and by 20 weeks, a fibrotic phenotype emerges, indicating that CD36 accelerates disease progression through early lipid accumulation and that CD36 overexpression in PTECs promotes inflammation and fibrosis [94]. These mechanisms highlight the role of various kidney cells in FA uptake abnormalities, linking kidney fibrosis and fatty acid toxicity to the development of CKD.

#### 3.2.2. Cholesterol Toxicity

Dysregulation of cholesterol metabolism is one of the hallmarks of CKD. Early studies showed that a high-cholesterol diet caused lipid deposition in rabbit kidneys [95]. Prospective cohort studies have also shown that familial hypercholesterolemia is linked to a heightened risk of CKD [96], highlighting cholesterol toxicity as a significant factor in CKD progression [97]. In CKD patients, increased SREBP expression accelerates cholesterol synthesis, leading to increased sterol O-acyltransferase 1 (SOAT1) activity and reduced neutral cholesterol ester hydrolase (NCEH) activity. Additionally, the nuclear translocation of ABCA1 and ABCG1 is inhibited, reducing their activity and preventing the elimination of excess cholesterol, causing cholesterol to accumulate in lipid droplets (LDs) [98,99].

Renal biopsies have revealed cholesterol accumulation in damaged podocytes of CKD patients [100,101]. Studies on diabetic nephropathy [102] and focal segmental glomerulosclerosis [103] have provided evidence that decreased ABCA1 expression in podocytes leads to significant cholesterol buildup and lipid droplet formation in the glomeruli. eGFR correlates positively with cholesterol metabolism, indicating that podocytes are key sites of cholesterol buildup. Cholesterol metabolism is disrupted in the TECs of fibrotic kidneys. In the TECs of fibrotic kidneys, excessive cholesterol induces endoplasmic reticulum stress and apoptosis [104], underscoring the role of cholesterol toxicity in kidney disease progression.

## 4. Key Mechanism of Ferroptosis in Advancement of CKD

**Figure 4:**
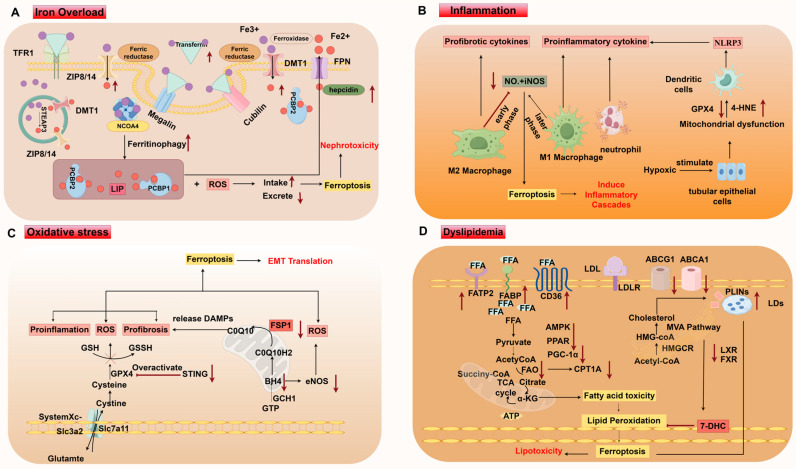
Mechanisms of ferroptosis in CKD. (**A**) Iron overload. (**B**) Inflammation. (**C**) Oxidative Stress. (**D**) Dyslipidemia. LDL low-density lipoproteins; LDLR LDL receptor; ABCG1 ATP-binding cassette subfamily G member 1; ABCA1 ATP-binding cassette subfamily A member 1; LXR liver X receptor; FXR farnesoid X receptor; PPAR peroxisome proliferator-activated receptor; PGC-1α peroxisome proliferator-activated receptor-gamma coactivator 1α; AMPK; AMP-activated protein kinase; SREBP sterol regulatory binding proteins; PLINS perilipins; LD lipid droplets; FFA free fatty acids; FAO fatty acid oxidation; CPTA1 carnitine palmitoyltransferase 1A; HMGCR 3-hydroxy-3-methylglutaryl-CoA reductase; 7-DHC 7-dehydrocholesterol; STING stimulator of interferon genes; DAMPs damage-associated molecular patterns; TECs tubular epithelial cells. This picture is drawn in Figure 2.

Renal parenchymal cell damage in CKD is often accompanied by micronutrient imbalances, ectopic lipid deposition, immune cell activation inducing inflammation, and an imbalance in the antioxidant system. These factors accelerate lipid peroxidation and promote renal parenchymal cell death. Ferroptosis, which involves these processes, plays a role in the pathogenesis of CKD, presenting a potential target for therapeutic intervention. (Figure 4).

### 4.1. Iron Overload Induces Nephrotoxicity

The kidneys meticulously regulate iron metabolism using various iron transport proteins and regulatory pathways throughout different segments of the nephron. Iron overload within the kidney can both cause and result from kidney injury, with ferroptosis linked to disruptions in iron metabolism, suggesting its role as a key etiological factor in CKD pathogenesis.

Iron overload induces nephrotoxicity [5]. In a 5/6 nephrectomy-induced CKD rat model, reducing iron intake disrupts iron metabolism and mitigates glomerular and tubular injury and fibrosis [105]. Elevated levels of TF and iron have been observed in the urine of patients with diabetic nephropathy and glomerulonephritis [106,107]. TFR1 and the Megalin–Cubilin complex are essential for iron uptake in PTEC, with differential transcriptional regulation affecting iron levels. Therefore, proximal tubules are capable of reabsorbing iron [108]. Additionally, ZIP8/14 promotes the involvement of both proximal and distal tubules in non-transferrin-bound iron (NTBI) uptake, accelerating iron absorption [109]. An increase in iron absorption could theoretically lead to intracellular iron overload, particularly in the distal tubules where ferroportin is absent, increasing susceptibility to ferroptosis.

Ferritin, abundant in the kidneys, affects the intracellular LIP. Therapeutic iron supplementation or FTH-specific knockout in renal macrophages can mitigate renal fibrosis and slow CKD progression [110,111]. This finding suggests that imbalances in FTH expression can lead to extremes in intracellular iron levels, either overload or deficiency.

PCBPs are essential regulators that collaborate with iron incorporated into ferritin [112]. A higher PCBP1 expression in tubules helps protect cells from ROS, whereas the specific mechanisms of PCBP2 are not fully understood [113]. PCBP1 potentially reverses alcohol-induced fibrosis in hepatic stellate cells by inhibiting proinflammatory and profibrotic signals. However, the precise roles of PCBPs in iron-induced ferroptosis during CKD need to be further investigated.

CKD patients often exhibit impaired hepcidin excretion due to decreased GFR. Inflammation increases hepcidin production, leading to high intracellular hepcidin levels and excessive Fe^2+^ storage, inhibiting heme synthesis, and disrupting iron utilization. This imbalance causes extracellular iron deficiency and intracellular iron overload. In models of adriamycin-induced focal segmental glomerulosclerosis, increased Fe^2+^ levels and elevated hepcidin levels drive tubular atrophy and interstitial fibrosis [114]. Similarly, ochratoxin A-induced renal toxicity involves intracellular iron accumulation driven by FPN downregulation, increased hepcidin, and disrupted iron homeostasis, leading to ferroptosis [115]. Taken together, these studies reveal a lurking link between iron overload-induced ferroptosis and CKD progression.

### 4.2. Inflammation

The increase in inflammatory markers suggests the onset of CKD, with key participants being cells of the innate immune response. Ferroptotic cells release DAMPs, triggering the innate immune system and sustaining the low-grade inflammatory response observed in CKD. The interplay between inflammation and ferroptosis in CKD is observed from an immune cell perspective.

Macrophages differentiate into proinflammatory M1 macrophages, which have high nitric oxide (NO•) and inducible nitric oxide synthase (iNOS) levels and are more resistant to ferroptosis, whereas anti-inflammatory M2 macrophages, which produce less iNOS and NO•, are more susceptible [116]. In CKD, early macrophage polarization is predominantly M1, with M1 macrophages producing ROS and inflammatory factors. Later, M2 macrophage levels increase, releasing pro-fibrotic factors like TGF-β. Understanding the dynamics of macrophage polarization and its impact on ferroptosis during CKD progression is crucial, but further research is needed to fully elucidate this relationship [117].

Ferroptosis in cancer cells can impair dendritic cell-mediated antitumor immunity [118]. The activation of inflammasomes in DCs is a key driver of tubular interstitial fibrosis and inflammation in the kidneys. In CKD models, hypoxic renal tubular epithelial cells exhibit characteristics of ferroptosis, such as mitochondrial dysfunction, reduced GPX4, and increased 4-HNE, driving inflammation and fibrosis through the activation of the NLRP3 inflammasome in CD1+ DCs of the myeloid cell population, leading to elevated levels of IL-1β and IL-18 [119]. In the tumor microenvironment, tumor-associated neutrophils (PMN-MDSCs) undergo spontaneous cell death due to ferroptosis, which limits T cell activity [120], and neutrophils accumulate during renal fibrosis [121]. In ACSL4 knockout mice, the ferroptosis inhibitor Fer-1 decreases neutrophil infiltration. In CKD, neutrophils may exacerbate the ferroptosis process by releasing inflammatory mediators and ROS, thereby promoting kidney damage. Ferroptosis-induced neutrophil death may further release inflammatory cytokines, creating a vicious cycle that intensifies renal inflammation and fibrosis [122]. In patients with hyperhomocysteinemia, B cell-derived antibodies are deposited in glomerular endothelial cells, increasing membrane phospholipids and promoting iron accumulation, which causes ferroptosis [123]. CD20 monoclonal antibodies and ferroptosis inhibitors such as liproxstatin-1 can effectively improve kidney injury [124].

In brief, persistent kidney injury in CKD releases many endogenous DAMPs, provoking immunocytes and triggering inflammatory cascades. Further research is needed to elucidate the relationships between different immune cells and ferroptosis in CKD.

### 4.3. Oxidative Stress

The kidneys, as a high-energy metabolic organ, are sensitive to oxidative stress-induced damage. High levels of oxidative stress are observed in the early stages of CKD and increase as the disease progression to ESRD [125]. ROS in the kidneys are primary generated by enzymes such as the mitochondrial respiratory chain and NADPH oxidase (NOX). In renal tubular epithelial cells, H₂O₂ leads to decreased GPX4 and GSH levels, increased iron content, and elevated lipid peroxide levels, triggering ferroptosis. Ferrostatin-1, a ferroptosis inhibitor, effectively counteracts this oxidative damage [126].Antioxidant pathways help safeguard renal tissue from oxidative stress-related damage, including ferroptosis, by suppressing synthesis, removing excess ROS, and repairing injuries. In the context of ferroptosis, the accumulation of excess iron in chronic kidney injury cells triggers the Fenton reaction, generating large amounts of ROS and catalyzing their spread, leading to severe oxidative stress. GPX4 is a key antioxidant molecule in ferroptosis, with mitochondrial GPX4 being the primary form that limits ROS production in the kidneys. In diabetic nephropathy models, inhibiting GPX4 ubiquitination reduces oxidative stress, slows ferroptosis, and improves kidney function [127]. In cisplatin-induced acute kidney injury model, mitochondria autophagy in renal cells helps remove intracellular ROS and protect the kidneys from ferroptosis by upregulating GPX4 [128]. Overactivation of the STING1 pathway depletes GPX4, thereby increasing oxidative stress and ferroptosis susceptibility [129]. GPX4 inactivation enhances the susceptibility of renal tissue to ferroptosis, possibly because the physiological functions and cellular characteristics of the kidney make it more prone to oxidative stress.

FSP1 collaborates with coenzyme Q10 to scavenge lipid radicals, and its absence increases ferroptosis sensitivity in renal tubular epithelial cells [130]. In the fibrotic stage, of CKD, ferroptotic renal tubular cells rupture and release DAMPs, accelerating ferroptosis in adjacent cells through oxidative stress and lipid peroxidation [131]. FSP1 prevents ferroptosis by reducing oxidized phospholipids, and has been shown to repair sepsis-induced ferroptosis caused by renal injury [132].

Tetrahydrobiopterin (BH4) stabilizes endothelial nitric oxide synthase (eNOS). A deficiency in BH4 uncouples eNOS, leading to increased production of inflammatory factors and ROS, which accelerates oxidative stress and fibrosis in CKD. Ferroptosis inhibitors such as Fer-1 counteract this effect by upregulating eNOS and reducing oxidative damage [133]. Mycotoxins, which impair mitochondrial coenzyme Q binding, also alter cell morphology in tubular epithelial cells, generate a large amount of ROS and worsening to CKD progression [134]. Abnormalities in antioxidant pathways further exacerbate ferroptosis in CKD, highlighting the complex interplay between oxidative stress, ferroptosis, and kidney fibrosis.

### 4.4. Dyslipidemia

Dyslipidemia in CKD disrupts renal energy metabolism, damages cellular membranes, and induces mitochondrial and endoplasmic reticulum stress. These effects lead to lipid peroxidation, triggering ferroptosis, which contributes to renal dysfunction and fibrosis. Moreover, ferroptosis has also been identified as a critical link between lipid metabolism disorders and CKD development.

#### 4.4.1. Abnormal Fatty Acid Metabolism

Fatty acid metabolism varies across kidney regions, with specific transport proteins and enzymes playing crucial roles in CKD-related dyslipidemia. Ferroptosis is closely associated with abnormal lipid metabolism, driven by disruptions in these proteins and enzymes.

The key molecules involved in FAO play an important role in ferroptosis, as they regulate the metabolism of fatty acids and directly or indirectly influence the occurrence and progression of ferroptosis. CPT1A, an enzyme that regulates long-chain fatty acid entry into mitochondria for β-oxidation, plays a central role. Inhibiting CPT1A induces ferroptosis in HK-2 cells [135], while upregulating CPT1A expression in diabetic mouse models alleviates ferroptosis and improves kidney injury [136]. Ferroptosis has been observed in calcium oxalate-induced TEC injury, where PGC-1α recruits NRF2 to the GPX4 promoter region, collaboratively activating GPX4 to delay cell death [137]. In kidney ischemia–reperfusion injury associated with ferroptosis, the inactivation of AMPK diminishes some of the resistance to ferroptosis [138], and using AMPK agonists to promote AMPK activation and NRF2 activation can prevent the occurrence of ferroptosis in diabetic nephropathy [139]. By regulating the activity of these molecules, the ferroptosis process in CKD can be intervened

The abnormal expression of lipid transport proteins in CKD also contributes to ferroptosis. CD36 is crucial for non-esterified fatty acid uptake in podocytes. Inhibiting CD36 reduces lipid buildup and ROS production [92,140]. CD36 exacerbates tubular injury in CKD by increasing inflammation, oxidative stress, and fibrosis in TECs [141]. In high-fat and high-glucose conditions, excess fatty acids transported by CD36 promote renal fibrosis in HK-2 cells [142]. CD36 also plays a role in the tumor microenvironment, mediating FA uptake by CD8+ T cells and inducing lipid peroxidation and ferroptosis [143]. In AKI, CD36 specifically binds to and degrades FSP1, exacerbating renal injury [144]. Increasing levels of soluble CD36 (sCD36) in the circulation and tissue indicate disease severity. Targeting CD36 can prevent renal fibrosis and reduce CKD progression [92]. CD36 modification effects vary by cell type, and the knockout of CD36 in macrophages retards interstitial fibrosis in obstructive nephropathy, whereas in proximal tubular cells, it mainly reduces proteinuria [145]. These findings underscore the role of CD36 in promoting lipid peroxidation to support ferroptosis during CKD.

FATPs (SLC27a1-6) transport long-chain fatty acids. FATP1, FATP2, and FATP4 are highly expressed in proximal renal tubular cells, with FATP2 being one of the most abundant [93,146]. Consequently, FATP2 is a significant factor in FAO impairment, tubular atrophy, and interstitial fibrosis. Inhibiting FATP2 decreases lipotoxicity in tubular cells and alleviates tubulointerstitial fibrosis [147]. Targeting FATPs for inhibition may play a critical role in regulating ferroptosis [148,149,150,151,152,153,154,155]. In the tumor microenvironment, where macrophages and CD8+ T cells take up lipids via CD36, the knockout of CD36 in CD8+ T cells in mice diminishes lipid peroxidation and inhibits ferroptosis [143,156,157]. The selective upregulation of fatty acid transport protein 2 (FATP2) in tumor-associated neutrophils increases AA uptake and induces ferroptosis [120,158]. Exploring FATP as a regulator of lipotoxicity-driven ferroptosis could be valuable.

FATP2 facilitates the uptake of PUFA and converts AA into AA-CoA. Lipidomic analysis revealed that FATP2 knockout cells have lower AA-PE levels, whereas ACSL4 knockout does not significantly affect AA-PE levels. FATP2 and ACSL4 independently provide substrates for peroxidation, with FATP2 potentially replacing or cooperating with ACSL4 in ferroptosis, influencing CKD development. In tumor neutrophils, the elevated intake of AA during differentiation is upregulated, and increased FATP2 activity leads to excessive lipid peroxides and worsens CKD through ferroptosis [158].

Fatty acid-binding proteins (FABPs) are membrane proteins that enhance the absorption of abnormal FA uptake in CKD patients. The overexpression of FABP3 (heart-type FABP) in podocytes is linked to increased fatty acid-induced lipotoxic effects [159]. Ferroptosis inducers such as RSL3 and FIN56 can upregulate FABP3 in tumor cells activated by hypoxia-inducible factor-1α (HIF1α) [160]. These findings indicate that FABP3 might be involved in ferroptosis. The deletion of FABP4 in obese mice reduces the synthesis of proinflammatory factors and inhibits FA breakdown [161]. Increased FABP4 levels in nondialysis CKD patients indicate its potential as a therapeutic target for reducing fibrosis [162]. In tumors, increased FABP4 enhances PUFAs, increasing ferroptosis [163]. L-FABP, or liver-type fatty acid binding protein, serves as a sensitive biomarker for CKD [164]. Higher levels of L-FABP have been associated with antioxidant properties and protective effects on renal function [165]. L-FABP binds long-chain fatty acids, facilitates lipid signaling, accelerates FA metabolism, and reduces inflammation, alleviating tubular interstitial injury [166]. Its role as an endogenous antioxidant in CKD underscores its potential as a target for inhibiting ferroptosis and providing renal protection.

#### 4.4.2. Abnormal Cholesterol Metabolism

Cellular cholesterol homeostasis is vital for renal lipid metabolism. The dysregulation of reverse cholesterol transport and hypercholesterolemia is commonly observed in various stages of CKD [100,167,168]. Numerous lines of evidence suggest that cholesterol plays an important role in ferroptosis. However, in HEK293T cells (human embryonic kidney 293 cells), cholesterol does not directly induce ferroptosis. Several genes involved in distal cholesterol biosynthesis have been identified as ferroptosis inhibitors. Specifically, 7-dehydrocholesterol (7-DHC), an intermediate in the distal cholesterol biosynthesis pathway, is converted to cholesterol by DHCR7. This conversion suppresses ferroptosis by preventing membrane lipid peroxidation. In renal ischemia-reperfusion injury models, targeting DHCR7 to increase 7-DHC levels in renal tissue reduces blood urea nitrogen and creatinine levels, mitigating renal injury and inhibiting ferroptosis [169].

Liver X receptor (LXR) and farnesoid X receptor (FXR) are key regulators of renal cholesterol synthesis. FXR and LXR overexpression in a diabetic nephropathy mouse model triggers AMPK-induced inflammatory signaling pathways, which mitigate lipid accumulation, inflammation, oxidative stress, and fibrosis [170]. FXR and RXR also upregulate key ferroptosis-regulating proteins, including FSP1, GPX4, and enzymes such as ACSL3, which contribute to antioxidant activity and ferroptosis defense [171]. In contrast, ferroptosis inducers increase sensitivity to ferroptosis by promoting cholesterol ester production through the ACSL4-SOAT1 pathway [172,173]. Additionally, GPX4 deficiency in macrophages reduces ABCA1 and ABCG1 expression, leading to increased modified low-density lipoprotein uptake. This suggests that these proteins may help resist ferroptosis in CKD [174].

PCSK9 increases low-density lipoprotein cholesterol (LDL-C) levels in the bloodstream [175]. In LDLR^+/−^ mice fed a high-cholesterol diet, PCSK9 worsens renal fibrosis [176]. Recent studies have linked PCSK9 to ferroptosis in abdominal aortic aneurysms [177]. PCSK9 affects ferroptosis by altering lipid metabolism, suggesting that it could be a potential therapeutic target for cholesterol-related diseases and ferroptosis.

#### 4.4.3. Lipid Droplet Accumulation

Lipid droplets (LDs) are cellular organelles responsible for this process [178]. They are involved in membrane transport, protein storage and degradation, signal transduction, and detoxification [179,180,181]. In CKD, LDs accumulate notably in podocytes and renal tubular cells, reflecting disruptions in lipid balance and LD quality and function [182,183]. LD deposition in fibrotic kidneys is closely linked to changes in FAO proteins, cholesterol intake, and lipid autophagy [184]. LD numbers increase considerably during ferroptosis [185]. Lipid degradation and autophagy break down LDs into FFAs through selective degradation mediated by molecular chaperones such as the PLIN family [186]. For example, PLIN5 suppresses lipotoxicity and ferroptosis in cardiomyocytes by modulating the PIR-NF-κB axis [187]. In sepsis-related acute kidney injury, lipid autophagy promotes ferroptosis and renal injury in tubular epithelial cells [188], although its specific role in CKD requires further investigation.

## 5. Specific Drug Targets for Ferroptosis in CKD

Ferroptosis plays a crucial in CKD progression. Pathogenic factors can stimulate ferroptosis through various mechanisms, making it a novel research target for CKD research. Ferroptosis inhibitors offer protective effects by reducing labile iron, preventing lipid peroxidation, and eliminating lipid peroxides, thereby providing anti-inflammatory and antioxidant benefits. Additionally, regulating abnormal lipid metabolism proteins and enzymes also can impede ferroptosis. This section summarizes recent advancements in CKD treatment through ferroptosis inhibition and related agents (Table 1).

Iron chelators such as deferoxamine (DFO), deferiprone (DFP), deferasirox (DFX), and ciclopirox (CPX) prevent ferroptosis by binding excess iron, inhibiting ROS and lipid peroxide production, and treating renal fibrosis [213,214]. In CKD rat models, DFX inhibits the TGFβ-Smad3 pathway, reducing inflammation and oxidative stress while attenuating renal fibrosis [189]. DFO alleviates renal fibrosis and iron accumulation in adenine-induced CKD models by modulating iron metabolism [190]. CPX lowers ferritin levels through autophagy, improving kidney function in polycystic kidney disease (PKD) mice [191]. A novel 3-hydroxypyridin-4(1H)-one chelates divalent free iron, scavenges ROS, and promotes cell repair in kidney injury [192]. In addition to the use of ferroptosis inhibitors, a low-iron diet in CKD mouse models can reduce redox-active levels, delaying the deterioration of renal function [215]. Activated carbon adsorbents also mitigate iron deposition, reducing renal injury and fibrosis resulting from ferroptosis [190].

Treatments targeting lipid peroxidation can be categorized into two main types: radical scavengers and enzyme-based inhibitors of lipid peroxidation [197,216]. Ferrostatin-1, liproxstatin-1, and their analogs are the most researched ferroptosis inhibitors easing fibrosis. Ferrostatin-1 is capable of inhibiting the 15LOX–PEBP1 complex, preventing ferroptotic PE oxidation in vivo [217]. Ferrostatin-1 inhibits the regulatory factor HIF-1α/HO-1 and reduces lipid peroxidation in diabetic mice [194,195,196]. Liproxstatin-1 mitigates inflammation and renal fibrosis in UUO and IRI mouse models [197].

ACSL4 is a key target for the enzyme-based inhibition of oxidation during ferroptosis, particularly in renal injury. The novel ACSL4-targeting inhibitor AS-252424 (AS), a furan-2-ylmethylene thiazolidinedione, effectively reduces swelling and damage in renal tubular cells, decreases oxidized phospholipid fatty acids and inflammation, and suppresses ferroptosis in AKI [198]. Dexmedetomidine downregulates ACSL4 expression, reducing ferroptosis-mediated inflammatory responses and protecting renal function [199]. XJB-5-131, a mitochondrial-targeted nitric oxide donor, exerts dual antioxidant effects by specifically inhibiting ferroptosis in TECs, alleviating injury and inflammation in renal ischemia-reperfusion, and slowing CKD progression [200]. Fisetin inhibits ACSL4-mediated ferroptosis in renal tubular cells, improving renal fibrosis in CKD mice [8]. Other ACSL4 inhibitors, such as thiazolidinediones, including rosiglitazone, pioglitazone, and troglitazone, can reduce ferroptosis and lipid peroxidation induced by RSL3 and GPX4 knockout, similarly reducing oxidative stress and inflammation in CKD to protect the kidneys [201,202,203]. Additionally, inhibiting LOX enzymes, such as baicalein, has also hindered the pathophysiology of AKI and CKD, reducing neutrophil infiltration and peroxide substrate production and decreasing ferroptosis in CKD [204,205]. NADPH oxidase 4 (NOX4) promotes the production of intracellular ROS, enhancing oxidative stress, lipid peroxidation accumulation, and ferroptosis. The natural flavonoid glycoside kaempferitrin inhibits NOX4-mediated ferroptosis, alleviates renal fibrosis and inflammation, and protects the kidneys [206]. Hederagenin inhibits ferroptosis induced by the TGF-β/Smad3 signaling pathway in renal tubular cells by reducing NOX4 expression and increasing GPX4 expression, thereby improving fibrosis in diabetic nephropathy mouse models [207].

Inhibiting the degradation of antioxidant factors has emerged as a potential therapeutic approach to halt radical chain reactions in various pathological conditions. The combination of melatonin and zileuton upregulates the AKT-mTOR-NRF2 signaling pathway, which inhibits ferroptosis and synergistically decreases fibrosis in UUO models [210]. Formononetin inhibits the Smad3-ATF3-SLC7A11 pathway, increases SLC7A11 and GPX4 expression, promotes NRF2 nuclear accumulation, and ameliorates renal fibrosis and ferroptosis [208]. Celastrol modulates NRF2 to upregulate GPX4, reducing intracellular iron accumulation and lipid peroxidation [211]. Vitexin activates the KEAP1-NRF2-OH-1 pathway, leading to increased GPX4 expression, the inhibition of lipid peroxidation and ferroptosis, and improvements in renal tubular injury, interstitial fibrosis, and inflammation [209].

Abnormal activities of transport proteins or enzymes involved in renal lipid metabolism can accelerate ferroptosis in CKD patients. Targeting these proteins or enzymes may help reduce the likelihood of ferroptosis and mitigate renal injury. Astragaloside IV (AS-IV) inhibits CD36 expression, reduces lipid ROS, downregulates ACSL4 and P53, and upregulates GPX4, reducing the risk of myocardial cell death in diabetic mice [212]. The FATP2-specific inhibitor lipofermata reduces AA accumulation and PGE2 production, thereby decreasing ferroptosis-related gene expression and slowing tumor progression [158]. Lipofermata also regulates profibrotic factor secretion and endoplasmic reticulum stress, improving renal fibrosis [147]. Canagliflozin influences lipid metabolism by upregulating the expression of the transcription factor FOXA1, which in turn increases CPT1A expression, promoting fatty acid oxidation and reducing ferroptosis in TECs in DN [136]. Similarly, empagliflozin prevents ferroptosis in diabetic nephropathy by promoting the AMPK-mediated NRF2 activation pathway [139]. However, the role of lipid metabolism enzymes in ferroptosis remains largely unexplored.

## 6. Frontiers and Prospects of Ferroptosis in CKD

Increasing evidence indicates a significant association between ferroptosis and CKD. This review systematically elucidates the mechanisms underlying the interplay between renal pathology and ferroptosis, providing a novel perspective on CKD. Ferroptosis contributes to CKD through abnormal iron deposition, ectopic lipid accumulation, and imbalances in antioxidant pathways, which lead to oxidative stress and inflammatory cascades. These conditions create an environment conducive to the progression of CKD. Timely and multifaceted interventions targeting ferroptosis could emerge as novel approaches for controlling CKD progression, alleviating kidney injury, and managing complications. However, current research remains at an early stage, with targeted clinical transition and development still in progress. Furthermore, this review is limited by its focus on classical ferroptosis regulatory mechanisms, and future research must address the following issues:

**Interaction with other types of cell death:** Understanding how ferroptosis interacts with other cell death mechanisms—such as autophagy, apoptosis, necrosis, cuproptosis, and disulfidptosis—is essential. This involves determining whether these interactions facilitate or impede CKD progression, which could unveil novel treatment opportunities.

**Interaction and transmission mechanisms between ferroptosis and abnormal lipid metabolism in CKD:** It is crucial to explore whether key fatty acid signals trigger ferroptosis during the process of abnormal lipid metabolism in CKD, including the pathways through which ferroptosis influences these conditions. Additionally, the ways in which ferroptosis propagates in renal parenchymal cells should be clarified.

**Biomarker identification:** Identifying specific biomarkers for ferroptosis could facilitate the early and accurate detection of CKD progression. The development of such biomarkers will be crucial for monitoring disease status and assessing the effectiveness of new treatments.

## 7. Conclusions

CKD remains a global public health issue with high morbidity and mortality rates, and persistent kidney fibrosis is a key factor leading to ESRD. Ferroptosis is closely linked to CKD. In this review, we systematically summarize the pathological mechanisms of ferroptosis in CKD and discuss potential therapeutic drugs to delay renal fibrosis, opening new avenues for future drug development.

In conclusion, the full extent of ferroptosis in the pathological process of CKD remains unclear. Despite the challenges, in-depth studies on the regulatory mechanisms of ferroptosis in CKD hold great potential for discovering effective biomarkers and therapeutic strategies. We firmly believe that focusing on ferroptosis research could bring new insights and directions for the diagnosis and treatment of CKD.

## Figures and Tables

**Table 1:** Molecular modulators of ferroptosis in CKD UUO, unilateral ureteral obstruction; IRI ischemia–reperfusion injury; DN, diabetic nephropathy; TECs, tubular epithelial cells, HO-1, heme oxygenase-1; AKI, acute kidney injury; UIR, unilateral renal ischemia-reperfusion; GSH, glutathione; GSSG, glutathione disulfide; NRF2, nuclear factor erythroid 2-related factor 2; GPX4, glutathione peroxidase 4; KEAP1, Kelch-like ECH-associated protein 1; PGE2, prostaglandin E2; PMN-MDSCs, polymorphonuclear myeloid-derived suppressor cells, PKD, polycystic kidney disease.

Types	Drugs	Target	Experiment Models	Cell Types	Mechanism	References
Iron chelator	Deferasirox	Ferrousion	5/6 nephrectomy mouse model	NONE	Inhibit oxidative stress and inflammation and activates the TGFΒ1-SMAD3 pathway	[189]
	Deferoxamine	Ferrousion	UUO/adenine- induced CKD mouse model	TECs		[190]
	Cicloprox	Ferrousion	PKD mouse model	Primary Human PKD Cyst-Lining Epithelial Cells	Ferritin degradation via ferritinophagy	[191]
	Novel 3 Hydroxypyri din-4(1H)- One	Ferrousion	NONE	cisplatin-induced AKI models	Reduce labile iron levels and scavenging radicals.	[192]
	Nuciferine	Ferrousion	Folic acid induced AKI	HK-2cells/HEK293T Cell	Inhibit theTLR4/PI3K/NF- κB pathway mitigates iron accumulation	[193]
ROS antioxidants	Ferrostatin-1	15LOX– PEBP1	*Pkd1^RC/RC^* mice db/db, UUO, IRI mouse model	Primary renal tubular epithelial cells	Inhibit inflammatory signal ing pathway, Reduce lipid peroxidation and kidney fibro sis	[194,195,196]
	Liprostatin-1	Lipidperoxi dation	UUO mouse model	HK-2 cells	Reduce the activation of pro-fibroblasts, lipid peroxidation, and iron deposition	[197]
Enzymatic lipid peroxidation	AS-252424	ACSL4	AKI mouse model	HK-2, and HEK293T cells	Decrease phospholipid fatty acids and blocks neutrophil infiltration	[198]
	Dexmdetomdine	ACSL4	I/R mouse model	HEK293T cells	Decrease inflammation	[199]
	XJB-5-131	ACSL4	I/R mouse model	NONE	Reduce inflammatory and lipid peroxidation	[200]
	Thiazolidinedi (rosiglitazone Pioglitazone troglitazone)	ACSL4	C57 mice	NRK-52E Cells and HK-2 cells	Reduce lipid peroxidation, redox stress and inflammation	[201,202,203]
	Fisetin	ACSL4	UUO/Adenine diet-induced mouse model	Tubular epithelial cells	Reduce iron, elevate the GSH and GSH/GSSG attenuate lipid peroxidation	[8]
	Baicalein	ALOX12	Cisplatin-induced AKI model	HK-2 cells	Inhibit ALOX12 reduce lipid peroxidation	[204,205]
	Kaempferitrin	NADPH oxidase 4	UUO mouse model	Primary tubular Epithelial Cells	Attenuate inflammation, fibrosis	[206]
	Hederagenin	NADPH oxidase 4	DN mouse model	HK-2 cells	Inhibit TGF-β/Smad3 signaling pathway	[207]
Inhibit the degradation of antioxidant factors	Formononetin	SLC7A11 and GPX4	UUO and FA mouse model	Primary tubular epithelial cells	Inhibit SMAD3/ATF3/SLC7A11 pathway and enhancement of antioxidant activity, reduce proinflammatory and profibrotic factors	[208]
	Vitexin	NRF2	UUO and UIR mouse model	HK-2 cells and NRK-49 F cells	Activate the NRF2/HO-1 pathway by inhibiting the KEAP1and of NRF2, increase GPX4 expression	[209]
	Melatonin and Zileuton	NRF2	UUO mouse model	HK-2 cells	Activate the AKT/mTOR/NRF2signaling pathway Promote the intracellular antioxidant	[210]
	Celastrol	GPX4	Cisplatin induced AKI mouse model	HK-2 cells	Inhibit NRF2 upregulated GPX4, reduce iron accumulation and lipid peroxidation	[211]
Enzymes in lipid metabolism	Astragaloside IV	CD36	Diabetic cardiomyopath mouse model	H9c2 cardiomyo cytes cell	Inhibit CD36 expression and decrease cellular lipid deposition, MDA, and lipid ROS production	[212]
	Lipofermata	FATP2	UUO mouse model	HK-2 cells, PMN-MDSC	Inhibit the expression of profibrotic factors Reduce arachidonic acid and PGE2 production	[147,158]
	Canagliflozin	CPT1A	Diabetic db/db mouse model	TECs under diabetic conditions	Improve FAO and attenuate ferroptosis of RTECs via FOXA1-CPT1A axis	[136]
	Empagliflozin	AMPK	Diabetic kidney disease mouse model	HK-2 cells,	Promote the AMPK-mediated NRF2 activation pathway	[139]

## Data Availability

No new data were created or analyzed in this study. Data sharing is not applicable to this article.

## Abbreviations

CKD chronic kidney disease; AKI acute kidney injury; DAMPs damage-associated molecular patterns; PUFAs polyunsaturated fatty acids; MUFAs monounsaturated fatty acids; ROS reactive oxygen species; TGF-β transforming growth factor-β; TBI transferrin-bound iron; NTBI non-transferrin-bound iron; TF transferrin; TFR transferrin receptor; TFR1/C transferrin receptor1/C; FATPs fatty acid transport proteins; FABPs fatty acid-binding protein; TECs tubular epithelial cells; TLR toll-like receptor; IL interleukin; IRI ischemia–reperfusion injury; HMGB1 high-mobility group box 1; UUO unilateral ureteral obstruction; DN diabetic nephropathy; Th T helper cells; FAO fatty acid oxidation; TG triglycerides; TNF tumor necrosis factor; MMP 9 matrix metalloproteinase 9; FGF-2 fibroblast growth factor 2; PDGF Platelet-Derived Growth Factor; DCs dendritic cells; CCL chemokine ligand; MCP monocyte chemotactic protein; 7-DHC 7-dehydrocholesterol; FA fatty acid; LDs lipid droplets; NOX NADPH oxidase.
